# Femtosecond Laser Engraving Promotes the Repopulation of Decellularized Human Articular Cartilage

**DOI:** 10.1155/term/2334978

**Published:** 2025-12-30

**Authors:** Conny Schneider, Johann Zehetner, Barbara Schädl, Matthias Domke, Claudia Keibl, Bernhard Rieder, Patrick Heimel, Anne Kleiner, Andreas Teuschl-Woller, Susanne Wolbank, Heinz Redl, Sylvia Nürnberger

**Affiliations:** ^1^ Ludwig Boltzmann Institute for Traumatology, the Research Center in Cooperation with AUVA, Donaueschingenstraße 13, Vienna, 1200, Austria; ^2^ Austrian Red Cross Blood Transfusion Service of Upper Austria, Krankenhausstraße 7, Linz, 4020, Austria; ^3^ Austrian Cluster for Tissue Regeneration, Vienna, Austria, tissue-regeneration.at; ^4^ Research Centre for Microtechnology, Vorarlberg University of Applied Sciences (FHV), Hochschulstraße 1, Dornbirn, 6850, Austria; ^5^ University Clinic of Dentistry, Medical University of Vienna, Sensengasse 2a, Vienna, 1090, Austria, meduniwien.ac.at; ^6^ Department Life Science Engineering, University of Applied Sciences Technikum Wien, Höchstädtplatz 6, Vienna, 1200, Austria, technikum-wien.at; ^7^ Department of Orthopedics and Trauma Surgery, Division of Trauma Surgery, Medical University of Vienna, Währinger Gürtel 18-20, Vienna, 1090, Austria, meduniwien.ac.at

**Keywords:** biocompatibility, biomaterial, cartilage regeneration, cell differentiation, decellularization, laser engraving

## Abstract

Decellularized articular cartilage of human origin presents itself as the most homologous filling material for focal cartilage defects. Yet, the full repopulation of the exceptionally dense collagen construct has never been achieved without providing host cells with artificially created migration paths into the matrix. Within this study, we examine the use of a femtosecond laser to engrave fine patterns into human articular cartilage before decellularization and GAG depletion (decell‐deGAG). Scaffolds were tested for decellularization success and mechanical behavior. Seeding tests were performed to assess biocompatibility and examine the performance in a simulated defect environment using an osteochondral plug model in vitro and in vivo in an ectopic nude mouse model. The composition and structure of the newly formed repair tissue and macrophage recruitment were observed via histology. The femtosecond laser was successful in engraving deep, fine structures into the matrix without the thermal damage found with other laser techniques. Engraving was also beneficial for decellularization success. The resulting decell‐deGAG scaffold featured a compressive modulus many times stronger than other biomaterials commonly used for cartilage regeneration and presents a defect filling material that is similar to the tissue it is meant to replace. Moreover, the incisions promoted the repopulation with therapeutically relevant cells. A favorable spatial environment inside the incisions facilitated the formation of repair tissue that mimics hyaline cartilage in composition and collagen orientation. Scaffolds were well‐integrated within simulated defects. Femtosecond laser–engraved cartilage poses an authentic defect filling material with cartilage‐like properties. When used in combination with cell seeding, it promotes the formation of differentiated repair tissue. Thus, the hereby presented biomaterial shows great potential in improving the repair of focal cartilage defects and reducing long‐term graft failures.

## 1. Introduction

The role of biomaterials for the repair of articular cartilage defects goes beyond merely distributing the cells within the defect. By a variety of spatial, mechanical, and biochemical cues, they can effectively guide the fate of seeded cells and the formation of repair tissue [1]. However, synthetic materials and collagen‐derived scaffolds currently used in clinics only loosely resemble native cartilage in spatial or chemical composition [2]. Thus, improved biomaterials may be a powerful tool to counteract the long‐term functional deterioration and high reoperation rates [3–7] often observed in the current state‐of‐the‐art treatments. Treatment failures are suspected to be caused by the formation of fibrous cartilage repair tissue unable to provide the specific biomechanical characteristics needed for articular cartilage [2, 8–10]. To guide the cells toward the formation of hyaline cartilage, it may be beneficial to take cues from nature and aim for a scaffold that mimics the composition and structure of the desired repair tissue.

Articular cartilage presents itself at the most homologous option and is known to provide chondrogenic cues that can counteract the dedifferentiation of chondrocytes and guide stem cells toward the chondrogenic lineage [11–13]. Therefore, decellularized articular cartilage as a homologous allogenic transplant has often been proposed as the most promising biomaterial for cartilage regeneration [14–18]. The repopulation by host cells, however, remains an ongoing challenge, as without further processing cellular ingrowth is limited to outer regions [17, 19–21].

Therefore, new approaches focus on creating space for cells to migrate into the otherwise intact cartilage matrix. A sandwich model with thin acellular cartilage sheets, stacked and seeded with chondrocytes, achieved a Young’s modulus close to native cartilage and preserved the original matrix orientation but disturbed the collagen continuation [22]. Incisions cut into the deep zone of articular cartilage with a scalpel offered a method to enhance the surface area with minimal disturbance to the Collagen type II architecture. Repopulation remained limited though, as the incisions mostly remained closed after implantation into the defect [23]. Holes pierced through the scaffold with a needle demonstrated successful repopulation with adipose‐derived stem/stromal cells (ASCs) in vitro [24, 25]. However, all these approaches involve manual cutting steps that are laborious and difficult to standardize and therefore unsuited for clinical application.

To generate space for cellular repopulation of cartilage in a more precise and standardized way, other studies feature the automatized option of laser‐generated channels [26, 27]. Cells are guided into the grafts, enabling more uniform tissue repopulation while having no negative influence on the mechanical properties. Commercial products such as ProChondrix demonstrate that laser‐modified cartilage matrices can be successfully translated into clinical applications. ProChondrix is a viable cartilage allograft that retains live donor chondrocytes and uses laser engraving to facilitate initial cell migration and integration. As a biological implant, it offers a natural source of matrix and cells and has shown promise in treating chondral defects in a one‐step procedure [28]. However, the presence of viable cells necessitates tight donor screening and strict cryopreservation protocols. In contrast, our approach aims to develop a cell‐free, decellularized scaffold to be repopulated with host cells. This strategy eliminates the risks associated with donor health and immunogenicity while enabling standardized production and broader clinical applicability. Because the scaffold does not rely on embedded viable donor cells, a more extensive microstructuring, such as the fine, deep laser engraving presented here, is required to support cell guidance, infiltration, and nutrient exchange.

In our previous study ([29], Figure 1(A)), we have refined this technique and added the novel concept of a laser‐engraved grid pattern, followed by decellularization and glycosaminoglycan (GAG) (decell‐deGAG) depletion. This laser pattern leaves the superficial zone intact, while enhancing the scaffold flexibility and cell–scaffold contact. The incisions have been shown to be suitable for the repopulation with therapeutically relevant cells, which were found to have migrated into the cartilage tissue up to 100 μm from the laser‐engraved edges. After 6 weeks in vivo, cells were most strongly differentiated at the very tip of the incisions, indicating that they benefited from chondrogenic cues in the confined environment.

Figure 1The use of a femtosecond laser allows for fine structures without Collagen type II alteration. (A) As established previously [29], laser‐engraved patterns can provide cells with access into deep regions of the scaffold. A CO_2_ laser generates V‐shaped incisions, where Collagen type II is altered within about 50 μm from the lasered edges. (B) Depending on the laser settings, lines engraved with a femtosecond laser can be spaced 250 μm (a; 1000 runs) or even 100 μm apart (b; 600 runs); in contrast to CO_2_ laser–engraved incisions, no Collagen type II alteration is visible on the immunostained slides; the μCT scan of a biopsy, 600 μm thick and 6 mm in diameter, engraved with 600 runs and 150 μm spacing (c, d) highlights the high regularity of the fine structures; edges for sample fixation during lasering, as seen on the CT scan, are cut off before implantation into the defect (c, image taken on a stereo microscope).

**Figure:**
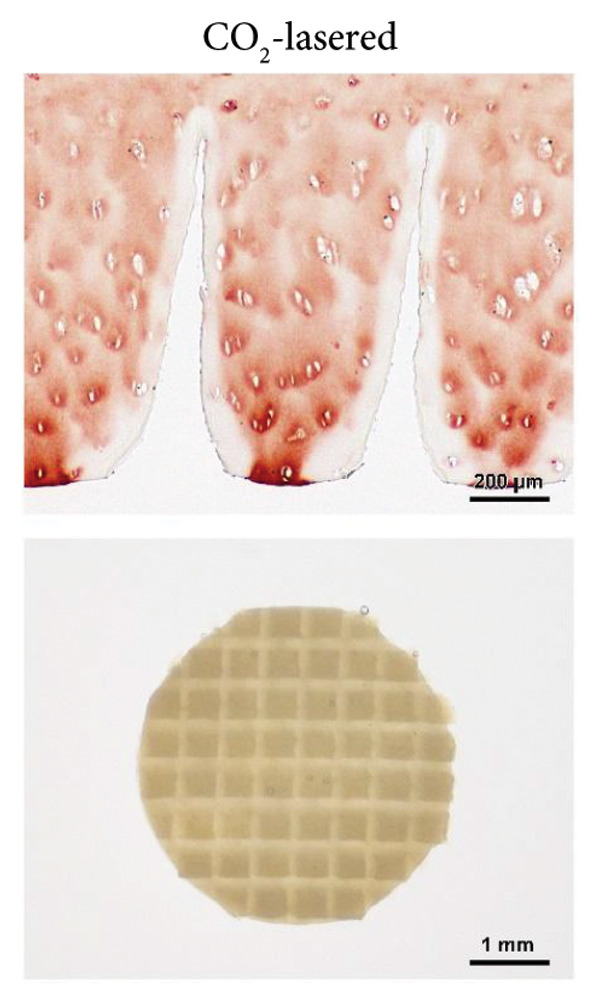
(A)

**Figure:**
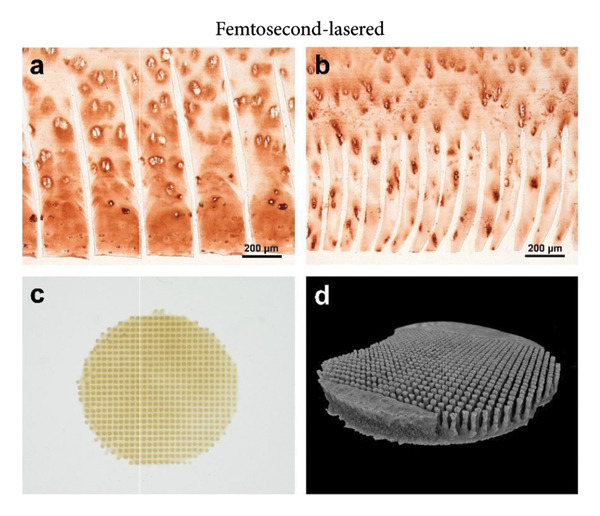
(B)

To enhance these chondrogenic cues as well as achieve a more homogeneous distribution of the cells within the scaffold, this study explores the use of a femtosecond laser as an alternative to previously applied CO_2_ laser engraving. While not necessarily superior in all parameters, femtosecond laser engraving offers fundamentally different physical characteristics, most notably, nonthermal ablation and higher incision precision, which may enable finer microstructuring of the matrix. Femtosecond lasers are already widely used in clinics for corneal surgery [30–33] and dental cavity preparation [34–37]. Their unmatched precision has been demonstrated in studies featuring nanosurgery of subcellular structures [38–40] or targeted modulation of single collagen fibers [41]. While with the CO_2_ laser, tissue ablation is largely thermal and may damage surrounding tissue, femtosecond lasers feature ultrashort pulses to induce the breakdown of molecules via photophysical processes with a minimal generation of heat. Targeted matrix disruption by a femtosecond laser has been shown to enable the repopulation of decellularized aortic valve scaffolds, successfully distributing the cells throughout the matrix [42]. The capacity to also ablate cartilage in a comprehensive way has been shown in a study assessing the use of different lasers to enhance the precision of microfracturing [43]. Thus, femtosecond lasers appear to be an optimal tool to generate deep, narrow incisions in a highly precise and reproducible manner, without thermal denaturation of the matrix observed in our previous study [29], which can compromise local tissue quality. By avoiding collateral damage, we expect that the femtosecond laser allows incisions to be placed more narrowly and uniformly, achieving a therapeutically relevant balance between sufficient matrix opening and preservation of collagen integrity, supporting scaffold functionality and cell viability.

The hereby presented scaffold fills the defect with a matrix most homologous to the lost tissue. We also expect the articular cartilage matrix, with its high surface area enhancing the extent of cell–scaffold interactions, to offer cues that promote a functional chondrogenic phenotype. These aspects make femtosecond laser–engraved cartilage a promising option to promote the formation of long‐lasting regenerative tissue of high quality.

We hypothesize that femtosecond laser ablation, due to its nonthermal, high‐precision mode of action, enables the creation of highly regular incisions in human articular cartilage without compromising the native matrix architecture. This could open the dense collagen network for efficient cellular repopulation while preserving the biomechanical and biochemical cues of the tissue.

To test this hypothesis, we asked whether femtosecond laser engraving can reliably produce fine, deep, and spatially controlled incisions and whether these modifications allow the accessibility and repopulation of decellularized cartilage scaffolds by chondrogenic cells.

## 2. Materials and Methods

### 2.1. Sample Harvest

With approval from the local ethics committee, human articular cartilage was obtained from macroscopically intact regions of femoral heads from patients undergoing total hip arthroplasty or hemiarthroplasty. Full‐thickness, noncalcified cartilage was dissected from the subchondral bone using a scalpel. Circular biopsies (8‐mm diameter) were produced with a punch, then washed, and stored in phosphate‐buffered saline (PBS) (Ca^2+^/Mg^2+^‐free, Lonza, Switzerland) supplemented with gentamycin and Amphotericin B (both Gibco, United States). For reseeding experiments, specimen thickness was standardized using a custom cutting device to 600 μm (for in vitro work and shallow defects) or 1 mm (for deeper defects).

### 2.2. Quantification of Chondron Spacing

To evaluate the spacing of chondrons in the deep zone of articular cartilage, the horizontal distance between chondrons was assessed on histological images of healthy human articular cartilage. The region of interest (ROI) was set to 400–1100 μm below the superficial zone to represent the main region where laser engraving takes place. For each chondron, the distance to its closest neighbor to the right was measured, determined by an axis in parallel to the biopsy’s surface plus a 22.5° angle upwards and downwards, respectively (amounting to a range of 45° to the right of the chondron). The measurements were then grouped into different distance categories, in 30 μm steps, to get an idea not only of the mean distance but also the distance distribution.

### 2.3. Laser Settings

Optimization of engraving parameters and imaging workflow was performed as described earlier for CO_2_ laser–engraved scaffolds [29], adapted here for femtosecond laser use. A Spectra‐physics Spirit 1040‐4‐SHG (Spectra‐Physics, United States) ultra‐short pulsed laser was used for engraving. The laser was operated at a wavelength of 520 nm and a pulse repetition rate of 100 kHz. A 100‐mm lens was used to focus the beam to a diameter of about 12 μm, measured at the 1/*e* ^2^ intensity level. The laser pulse energy was constantly set to 7 μJ, resulting in a peak fluence of 12.4 J/cm^2^.

During setting optimization, full‐thickness cartilage biopsies were used. Parallel lines were engraved with varying spacing, 400–1000 runs and a constant speed of 500 mm/s. The effects were evaluated macroscopically and via histology.

Based on the observations made during setting optimization, cartilage biopsies were engraved with a checker pattern (“grid pattern”), 400 runs with 150‐μm spacing for 600‐μm thick samples and 1000 runs with 250‐μm spacing for 1‐mm thick samples, for mechanical testing and reseeding. Stereo microscopy and μCT imaging were used to observe the regularity of the pattern. μCT imaging was performed on a SCANCO μCT 50 (SCANCO Medical AG, Switzerland), with 1% osmium tetroxide and 0.07% ruthenium hexamine trichloride (RHT) as contrast agent. Scans were performed at a tube voltage of 90 kVp and a current of 88 μA and a 0.5‐mm Al Filter. 1000 projections per 180° were exposed for 900 ms and reconstructed to an isotropic resolution of 4 μm. 3D Volume rendering was created using FIJI [44] and Drishti [45].

### 2.4. decell‐deGAG

Laser‐patterned cartilage underwent decellularization with selective matrix modification (“decell‐deGAG”) as previously described [16, 29].

In brief, for devitalization, four freeze/thaw cycles (−20°C/room temperature) were applied, twice frozen dryly and twice frozen submerged in hypotonic buffer (10 mM Tris‐base, Promega, United States, pH 8.0 adjusted with HCl). Samples were then incubated overnight in 0.1‐M HCl (Roth, Switzerland), followed by overnight pepsin treatment (1 mg/mL; Sigma, P7012, in 0.5 M acetic acid, Merck, Germany) to selectively deplete matrix components, and finally, 0.1‐M NaOH for 6 h to roughen cut edges. All steps were conducted at 37°C under continuous shaking, with extensive PBS washes between treatments.

### 2.5. Mechanical Compression Test

Mechanical compression analysis was performed on biopsies of native articular cartilage (8‐mm diameter, 1‐mm thickness) and laser‐engraved scaffolds, with or without decell‐deGAG on a Zwick BZ2.5/TN1S uniaxial testing machine (Zwick GmbH & Co. KG, Germany), equipped with a 50‐N load cell and a custom‐made unrestrained compression set. Specimens were equilibrated in PBS for 24 h before testing, and then placed in a well matching the sample diameter to emulate a defect bed. After a 50‐mN preload, samples were compressed to 80% deformation at 100 μm/min. The compressive modulus was calculated between 17% and 20% deformation. Clinically used implants Chondro‐Gide (Geistlich, Switzerland) and Hyalograft (FIDIA Advanced Biopolymers, Italy) served as controls.

### 2.6. DNA and GAG Quantification

Quantitative analyses of DNA and GAGs were carried out on scaffolds of both thicknesses (600 μm and 1 mm) after laser engraving alone, decell‐deGAG treatment alone, and the combined process used for the final scaffold, as well as untreated human articular cartilage as a control. Quantification followed the same analytical protocol as established in our earlier study [29]. Each condition comprised six biological replicates.

Samples were enzymatically digested with papain according to a protocol adapted from Kim et al. [46]. For DNA quantification, the samples were then treated with RNase (Sigma‐Aldrich, United States) for 30 min before CyQUANT GR dye from the CyQUANT Cell Proliferation Kit (Life Technologies, United States) was added. Fluorescence was read at 480‐nm excitation/520‐nm emission in 96‐well plates (Greiner, Austria) using a TECAN Infinite 200 plate reader (Tecan Group AG, Switzerland). Each sample was measured in duplicate, and DNA content was calculated by linear regression against a standard curve (DNA sodium salt from calf thymus, Sigma Aldrich, United States).

For GAG quantification, the dimethyl methylene blue (DMMB) assay was used as described in [47]. Digested samples were diluted with PBS‐EDTA, DMMB (Sigma Aldrich, United States) was added, and the resulting metachromatic absorbance shift was recorded at 530 nm and 590 nm. GAG concentrations were derived by linear regression to a chondroitin sulfate standard curve (Sigma‐Aldrich, United States).

### 2.7. Cell Culture

To minimize donor‐to‐donor and passage variability, immortalized human ASCs (ASCs‐TERT1) (Evercyte GmbH, Austria) were used as the ASC source and expanded in EGM‐2 (Lonza, Switzerland, a lab‐standard proliferation medium previously verified to preserve chondrogenic potential), with passaging at ∼70% confluence.

Human articular chondrocytes (hACs) were harvested from femoral heads of five donors (mean age 55 years) undergoing hip arthroplasty with the approval of the local ethical board. Cartilage was digested sequentially in series of enzymes: 1‐h incubation in hyaluronidase solution (0.1%, Sigma‐Aldrich, United States in DMEM, Gibco, United States), 0.5 h in pronase solution (0.1%, Roche Switzerland in DMEM), and overnight incubation in a collagenase/papain mix (200 U/mL collagenase, Gibco, United States, and 1 U/mL papain, Sigma‐Aldrich, United States, in DMEM). Cells were expanded in chondrocyte medium (DMEM supplemented with 10% FCS, PAN‐Biotech, Germany; 10‐mM HEPES and 2‐mM L‐glutamine, both Gibco, United States; 50‐μg/mL ascorbate 2‐phosphate and 5‐μg/mL insulin, both Sigma‐Aldrich, United States; and 2‐μg/mL Amphotericin B and 100‐μg/mL gentamycin). hAC were cryopreserved at subconfluency, thawed prior to experiments, and allowed to adapt to culture for 4 days before seeding onto the scaffold. Cells were pooled to reduce donor variability and restrict the number of animals in the subsequent in vivo experiment.

All cells were incubated at 37°C and 5% CO_2._

The purpose of including these cell types was not to compare their performance but to demonstrate scaffold compatibility with cell sources that are clinically relevant for cartilage repair.

### 2.8. Differentiation In Vitro

The in vitro reseeding tests were designed to reflect the subsequent in vivo experiment. Cells were seeded onto the scaffold in either mono or coculture, at a ratio of 75% ASC and 25% hAC. The time points and media vary between groups as ASC undergoes a precultivation period prior to implantation, which has been shown to be efficient in previous experiments [29, 48].

Biopsies with a diameter of 4 mm (reflecting the diameter of the defect in the in vivo experiments) were prepared from laser‐engraved scaffolds, 600 μm in thickness, and transferred to 1.5‐mL screw cap vials. After preincubation in DMEM +20% fetal calf serum at 37°C for 2 h, each scaffold was seeded with 0.25 ∗ 10^6^ cells in 1 mL EGM‐2 (for ASC) or chondrocyte expansion medium (coculture and hAC). Incubation took place at 37°C under continuous vertical rotation of 60 rpm around an internal as well as external axis for five (ASC) or 2 weeks (coculture and hAC); afterward, the samples were cultivated statically. Medium was replaced three times per week.

Scaffolds seeded with ASC were switched to Lonza chondrogenic differentiation medium (Lonza, Switzerland) supplemented with 10‐ng/mL BMP‐6 and TGFβ‐3 (both R&D systems, United States) 1 week after cell seeding, a period that had been shown to be beneficial for cell attachment in the dynamic environment. After two further weeks, medium was changed to chondrogenic differentiation medium (DMEM‐hg, Lonza, Switzerland, supplemented with 5‐mg/mL insulin, 5‐mg/mL transferrin, and 5‐ng/mL selenous acid via an ITS‐premix, Gibco, United States; 0.1‐mM dexamethasone, 0.17‐mM ascorbic acid‐2‐phosphate, 1‐mM sodium pyruvate, and 0.35‐mM proline, all Sigma‐Aldrich, United States; 2‐mM L‐glutamine, Gibco, United States; and Pen/Strep, Lonza, Switzerland, as described by Hennig et al. [49]) supplemented with 10‐ng/mL BMP‐6 and TGFβ‐3 for economic reasons. To reflect the extended cultivation period of ASC in subsequent experiments, samples were harvested 9 weeks after reseeding (6 weeks after the switch to self‐mixed chondrogenic differentiation medium).

For scaffolds seeded with hAC alone or in coculture with ASC, the medium was switched directly to chondrogenic differentiation medium, supplemented with 10‐ng/mL BMP‐6 and TGFβ‐3, 2 days after seeding. They were harvested 6 weeks after reseeding.

All scaffolds were subjected to histological examination, as described in Section 2.10.

For comparison, CO_2_ laser–engraved cartilage scaffolds and a commercially available Collagen I/III scaffold (Chondro‐Gide, Geistlich, Switzerland, an acellular collagen matrix commonly used for matrix‐assisted chondrocyte implantation procedures) were included as controls and seeded with the same cell types under identical cultivation conditions.

### 2.9. Osteochondral Plug Model In Vivo

To examine the performance of the scaffold in vivo in an articular cartilage defect environment, reseeded scaffolds were implanted into a simulated chondral defect in osteochondral plugs (as described by Schüller et al. [50]) and cultivated in a nude mouse model. The experimental setup and implantation procedure were performed as in our earlier study [29] to allow for comparison of the different laser‐engraved scaffold types.

Osteochondral plugs of 1 cm in diameter were harvested from the tibial plateau and the femoral condyles of bovine knee joints obtained from the local slaughterhouse. Full‐thickness cartilage defects, reaching down to the osteochondral interface, were prepared using a 4‐mm biopsy punch.

Scaffolds of 4 mm in diameter were seeded based on their thickness: 600‐μm thick scaffolds received 250,000 cells, and 1‐mm thick scaffolds were seeded with 500,000 cells, to ensure consistent cell density per scaffold volume. Thin scaffolds (*n* = 4 for ASC and chondrocyte groups, *n* = 3 for coculture) were used for shallower defects, while thick scaffolds (*n* = 2 for ASC and chondrocyte groups, *n* = 3 for coculture) were used to fill deeper defects.

ASCs alone were seeded 3 weeks prior to implantation and cultivated under continuous rotation in EGM‐2 for 1 week followed by Lonza chondrogenic differentiation medium supplemented with 10‐ng/mL BMP‐6 and TGFβ‐3 for 2 weeks before implantation.

hAC and cells in coculture were seeded in chondrocyte expansion medium, 1/3 of the cells directly onto the scaffold in a small volume and 2/3 into the defect. Samples were implanted into the defect immediately after reseeding.

In addition, CO_2_ laser–engraved cartilage scaffolds seeded with the respective cell types were implanted under the same conditions and analyzed as controls.

Scaffolds were fixed into the defect via superficial application of fibrin sealant (ARTISS, Baxter, USA). Plugs were cultivated submerged in chondrocyte expansion medium at 37°C and 5% CO_2_ overnight, followed by subcutaneous implantation in 10‐weeks‐old female Naval Medical Research Institute (NMRI) nude mice (Charles River, Sulzfeld, Germany). The NMRI mouse is an athymic breed allowing the implantation of nonautologous cells without rejection.

The experimental protocols were approved by the City Government of Vienna (Animal Use Permit No: MA58/982788/2015/13) prior to the study, in accordance with the Austrian law and the Guide for the Care and Use of Laboratory Animals as defined by the National Institute of Health (revised 2011). Surgeries were performed as previously described [29, 48]: Mice were anesthetized with 2%–3% isoflurane (AbbVie, Austria) and two paramedian incisions were made on the left and right sides of the back. The osteochondral plugs were placed in subcutaneous skin bags, one per incision, which were then closed with 6‐0 absorbable monofilament sutures (Monosyn, B Braun, Spain). All mice received 1‐mg/kg meloxicam (Metacam, Boehringer Ingelheim, Germany) orally 2 hours before surgical intervention and for 4 days (S.I.D.) postoperatively to alleviate pain and discomfort. After 6 weeks, the animals were sacrificed by using deep inhalation anesthesia with isoflurane followed by cervical dislocation. The osteochondral plugs were retrieved and subjected to histological examination.

In order to quantify the number of laser incisions bearing differentiated neo‐cartilage tissue, each single incision was evaluated. On histological paraffin sections of all six samples per group, the number of Collagen type II–positive incisions was counted as either evenly differentiated or differentiated with a “tip effect” (differentiation strongest deep inside the incisions) and compared with the number of total incisions and evaluated separately for hAC, ASC, and cells in coculture.

### 2.10. Histological Examination

Samples were immersed in 4% neutral buffered formalin overnight for fixation, followed by thorough rinsing in PBS and dehydration through a graded ethanol series. Osteochondral plugs were subsequently decalcified in USEDECALC solution (Medite, Germany) for 5 weeks. Both the cartilage biopsies and the decalcified plugs were bisected and embedded in paraffin via xylol (Roth, Switzerland). Serial sections of 3–4 μm thickness were cut using a rotary microtome (MICROM HM355S, Thermo Fisher Scientific, United States). Depending on the specific analytical purpose, different histological and immunohistochemical stains were employed.

Recellularization was visualized using AZAN staining, where the collagen matrix appears blue and viable cells red. The overall matrix composition was assessed using Alcian blue staining (0.3% at pH 2.5) with Sirius red counterstaining to identify GAG‐rich areas. Collagen type II was detected by immunostaining with antibodies against either newly formed (Clone 2B1.5) or total Collagen type II (Clone 6B3, both Thermo Fisher Scientific, United States). The presence of macrophages was verified through CD68 immunostaining (Abcam, Great Britain).

For immunohistochemical procedures, endogenous peroxidase and alkaline phosphatase activity were blocked using BLOXALL (Vector Labs, United States), and antigen retrieval was performed with pepsin (pH 2). Primary antibodies were applied for 1 hour at room temperature at dilutions of 1:100 for Collagen type II and 1:200 for CD68. As a secondary antibody, the polymer labelled system Bright vision poly HRP (VWR, United States) was incubated for 30 min at room temperature. A peroxidase substrate kit (NovaRED, Vector Labs, United States) was used for the development of the color reaction and hematoxylin for counterstaining.

For quantitative evaluation, every laser incision visible on the paraffin sections was examined for Collagen type II deposition. Incisions showing staining concentrated at the incision tip were categorized accordingly, and their frequency was compared to the total number of Collagen type II–positive incisions. This analysis was performed across all six samples per group and evaluated separately for scaffolds seeded with hAC, ASC, and cells in coculture.

### 2.11. Quantification of Macrophages

To assess the presence of CD68‐positive cells in defects following in vivo cultivation, quantification of the CD68‐positive area within the incisions was performed via a script using FIJI [44]. The ROI was manually selected to exclude the scaffold matrix. Two regions of interest were defined per defect. Via a color threshold, the script segmented positive and nonpositive areas and calculated the ratio of positive areas to the total area. Data were displayed as scatter plots with mean and standard deviation.

### 2.12. Statistics

The program GraphPad Prism (Version 6) was used for statistical analysis, and the normal distribution of the data was assessed by the Shapiro–Wilk test. The assumption of normally distributed values was rejected, thus subsequent tests on quantitative data of mechanical testing, DNA and GAG content, and presence of macrophages were performed with Mann–Whitney U tests. Data were displayed as scatter plots with mean and standard deviation.

### 2.13. Data Sharing

The raw datasets generated within this study are available on request from the corresponding author.

## 3. Results

Within this study, a femtosecond laser was used to make a dense matrix accessible for repopulation with host cells. Lines (for laser settings optimization) and finally grid (for mechanical testing and reseeding) patterns were engraved into human articular cartilage from the deep zone upwards, leaving a backbone (superficial zone) structurally intact. The scaffolds were subjected to decell‐deGAG prior to reseeding and tested for their mechanical characteristics and the behavior of seeded cells under chondrogenic conditions in vitro. Finally, the performance of the femtosecond laser–engraved decell‐deGAG scaffolds as defect filling material was demonstrated in an osteochondral plug model in vitro and in vivo.

### 3.1. Quantification of Chondron Spacing

In order to determine the line spacing needed to allow a cell spacing similar to the natural conditions, the distance of chondrons in the deep zone of articular cartilage was measured on histology slides.

**Figure 2:**
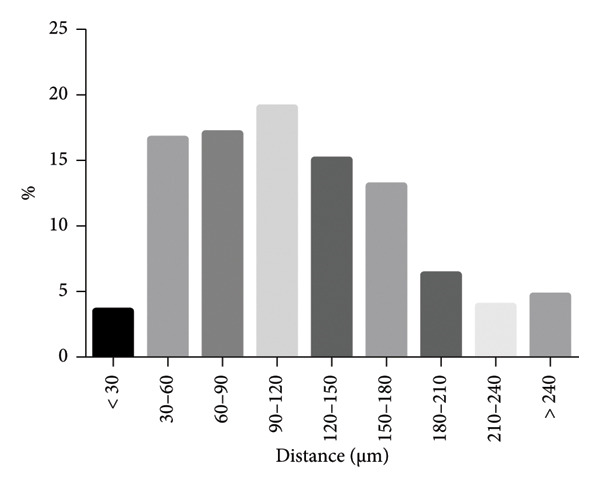
Distance of chondrons in the deep zone of human articular cartilage analyzed from histological images. A distance distribution reveals the most commonly occurring distance to the closest chondron to be in the range of 90–120 μm, and more than 3 out of 4 of the measured distances to lie within the range of 30 and 180 μm.

In total, 251 chondrons were evaluated. A mean distance of 118 μm was determined; however, the standard deviation of 60.1 μm highlighted a broad variety of distances. Thus, values were grouped into 30‐μm steps to visualize the frequency of various distances (Figure 2). The most commonly occurring distance to the closest chondron was shown to be in the range of 90–120 μm. The majority (81%) of all measured distances were between 30 and 180 μm long, 51% between 60 and 150 μm. We thus aimed for an incision spacing of 100 μm to match this distance.

### 3.2. Laser Settings

Different settings were tested to determine the optimal amount of runs and line spacing to obtain incisions as deep as possible while keeping our target line spacing of 100 μm in order to greatly enhance the surface area and distribute the cells more evenly throughout the scaffold.

Macroscopic evaluation, histology, and μCT revealed the tissue removal by the fs laser to be well focused and spatially confined, leading to deep narrow incisions with a constant width of about 30 μm. This allowed for a close spacing of the incisions and left cartilage structures of equally regular width. A line spacing of down to 100 μm was shown to be feasible for up to 600 runs. More runs negatively affected the stability of the remaining cartilage sheets. The number of runs determined the incision depth, with 650 μm at 600 runs and 1000 μm at 1000 runs (Figure 1(B)). Immunohistochemistry revealed no changes in the structure and staining intensity of Collagen type II.

To obtain the grid structures featured on the final scaffolds, orthogonally crossed lines were engraved, resulting in pillars with high regularity, as observed via μCT and a stereo microscope. The scaffold thickness was standardized to match the defects of different depths. Samples with grid structures were engraved with slightly greater spacing to ensure that stability was maintained. Biopsies of 600 μm in thickness were engraved with 400 runs and a spacing of 150 μm, 1‐mm thick biopsies with 900 runs and a spacing of 250 μm. The final scaffold featured a highly regular topography with deep incisions, as could also be observed in 3D using micro computed tomography (Figure 1(B)). In addition, supporting Figure 1 provides a dynamic 3D rendering of the μCT scan that offers a comprehensive view of the intricate grid pattern and deep incisions, highlighting the potential of a femtosecond laser to create fine, regular topography with high precision.

For comparison with CO_2_ laser engraving, selected parameters such as incision morphology and matrix damage were included from our previous study [29]. A comparative overview of key features of CO_2_ and femtosecond laser engraving is summarized in Table 1.

**Table 1:** Comparison of CO_2_ laser‐engraved and femtosecond laser‐engraved human articular cartilage scaffolds.

	CO_2_‐lasered cartilage (from [29])	Femtosecond‐lasered cartilage
Incision depth	900 μm (for 1‐mm thick cartilage), 300 μm (for 400 μm thick)	900 μm (for 1‐mm thick cartilage), 500 μm (for 600 μm thick)
Incision width at the surface layer	200 µm	20 µm
Minimum spacing	500 μm (for 1 mm thick), 300 μm (400 μm thick)	250 μm (for 1 mm thick), 100 μm (for 600 μm thick)
Cell ingrowth into the scaffold matrix	Observed in vitro and in vivo	Observed in vitro and in vivo
Ingrowth depth into the scaffold matrix	50 µm	50 µm
Incision interface	Collagen type II altered within 50 μm from the lasered edges	Collagen type II immunoreactivity intact
Compressive modulus	∼15% of native cartilage	∼15% of native cartilage
DNA content after decell‐deGAG (in 1‐mm thick samples)	41 ± 3.8 ng/mg	28 ± 3.7 ng/mg
GAG content after decell‐deGAG (in 1‐mm thick samples)	13 ± 2.0 μg/mg	14 ± 2.4 μg/mg

*Note:* Data for CO_2_‐lasered cartilage were taken from our previous study [29], while values for femtosecond‐lasered cartilage were obtained in the present work. The table summarizes incision geometry, spacing limits, matrix integrity at the incision interface, cellular ingrowth behavior, and biochemical and mechanical properties after decellularization and targeted GAG depletion (decell‐deGAG).

### 3.3. Mechanical Compression Test

The impact of femtosecond laser engraving and subsequent decell‐deGAG treatment on mechanical characteristics was examined via compression analysis. To bring the findings into context with the current clinical situation, commercially available scaffolds served as controls.

The stress–strain curve showed that untreated cartilage reached 80% deformation at a load of 720 kPa, while laser engraving reduced the stiffness to 300 kPa. The decell‐deGAG procedure caused further loss in stiffness, reaching 80% deformation at 90 kPa (Figure 3).

Figure 3Influence of laser engraving and matrix pretreatment on compressive stiffness and modulus of human articular cartilage compared to currently used materials. (a) While untreated cartilage reached 80% deformation at a load of up to 720 kPa, laser‐engraved grid patterns reduced the stiffness to a maximum of 300 kPa without and 90 kPa with subsequent decell‐deGAG procedure. (b) The compressive modulus (between 17% and 20% compression) of laser‐engraved cartilage is reduced to 60% compared to native cartilage. After matrix pretreatment, the final scaffold retains 16% of its native compressive modulus, many times stronger than the commercially available controls; data shown as mean ± SD, *n* > 3; ^∗^ *p* < 0.05 and ^∗∗^ *p* < 0.01.

**Figure:**
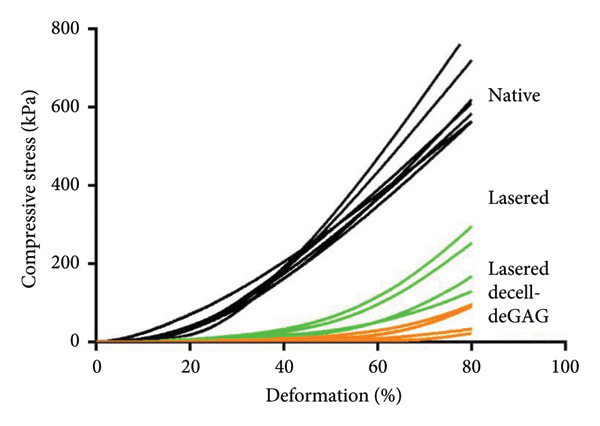
(a)

**Figure:**
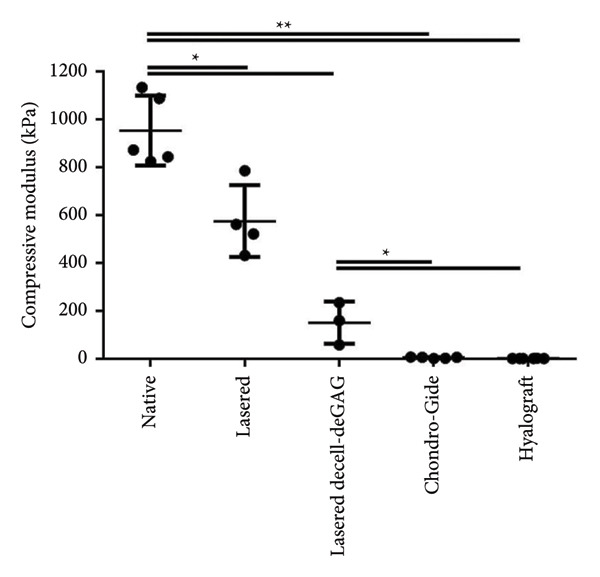
(b)

Calculated at a physiologically relevant compression of 17%–20% deformation, compressive modulus was the highest for untreated cartilage (953 ± 131 kPa), while laser‐engraved cartilage retained about 60% of its native compressive modulus (576 ± 130 kPa) and laser‐engraved decell‐deGAG cartilage about 15% (152 ± 71.9 kPa). The commercially available control scaffolds featured moduli of below 1% of native articular cartilage (Chondro‐Gide 6.16 ± 2.33 kPa; Hyalograft 2.56 ± 0.19 kPa). Thus, laser‐engraved decell‐deGAG cartilage remains 20 to 50 times stronger than any of the tested control scaffolds, which are currently used in the clinics (Figure 3).

### 3.4. DNA and GAG Content

**Figure 4:**
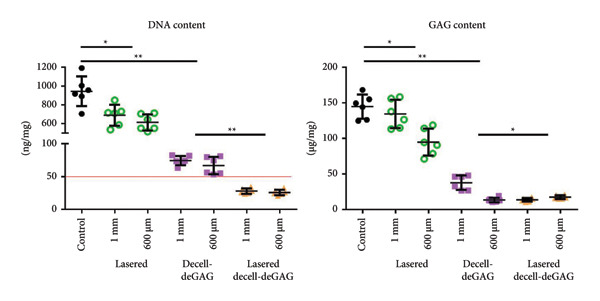
The influence of laser engraving and decell‐deGAG treatment on the DNA and GAG contents of articular cartilage. Laser engraving alone already leads to a reduction of the DNA content. The decell‐deGAG protocol alone greatly reduces the DNA content of unlasered samples, yet leaves them with more than 50‐ng/mg DNA, the commonly accepted threshold for decellularized tissue. Combined laser engraving and decell‐deGAG treatment leads to a DNA content below 50 ng/mg. 600‐μm thick samples reach their minimum after decell‐deGAG treatment alone, while 1‐mm thick samples benefit from previous laser engraving. *n* = 6, ^∗^ *p* < 0.05, and ^∗∗^ *p* < 0.01.

Biochemical assays were performed to assess the success of the decell‐deGAG process, as well as to determine the influence of laser engraving on its efficacy. Laser engraving alone already influenced the DNA content, reducing it from 945 ± 145 ng/mg dry weight to 688 + 102 and 613 + 78 ng/mg, respectively, for 1‐mm and 600‐μm thick samples (Figure 4). Decell‐deGAG treatment alone reduced the DNA content to 74 + 6.5 (1 mm) and 67 + 12 ng/mg (600 μm). The combination of laser engraving followed by decell‐deGAG treatment led to a final DNA content of 28 + 3.7 (1 mm) and 26 + 3.8 (600 μm) ng/mg dry weight. The GAG content was reduced from 145 + 15 μg/mg dry weight to 14 + 2.4 (1 mm) and 17 + 2.5 (600 μm) μg/mg after laser engraving and decell‐deGAG treatment.

### 3.5. Differentiation In Vitro

To examine the chondrogenic differentiation of therapeutically relevant cells on femtosecond laser–engraved scaffolds, hACs (from donors of advanced age), ASC‐TERT1, or a coculture thereof were seeded and cultivated in the presence of chondrogenic growth factors (10‐ng/mL BMP‐6 and TGFβ‐3). Cultivation lasted for six (hAC, coculture) or nine (ASC) weeks to account for the precultivation (chondrogenic prestimulation) period of ASC prior to implantation in the in vivo experiment.

**Figure 5:**
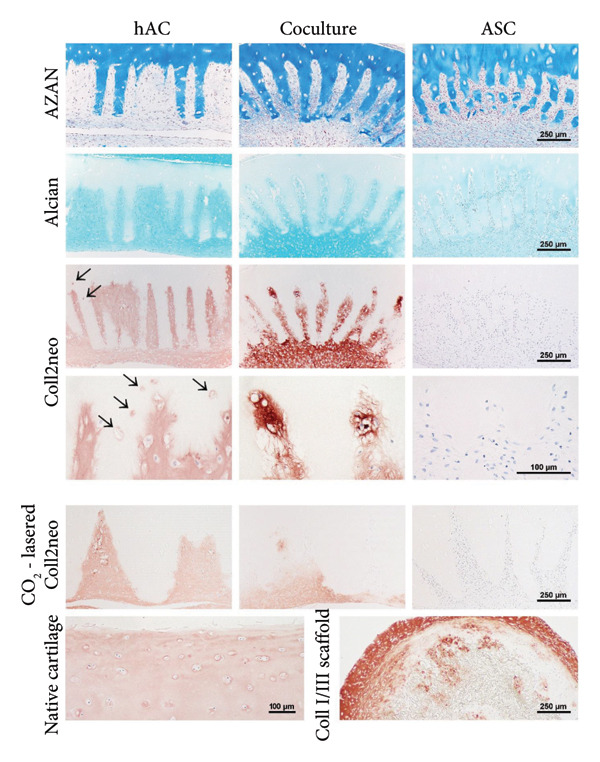
Repopulation and chondrogenic differentiation of ASC, hAC, and a coculture on the fs‐lasered articular cartilage in vitro. AZAN staining reveals the incisions to be densely populated by seeded cells of all types; scaffold contraction occurred in the ASC and the coculture group; Alcian blue staining of GAG and Collagen type II immunostaining are highly positive for the chondrocyte and the coculture group, while ASC alone remained undifferentiated; in the chondrocyte group, Collagen type II producing cells have invaded empty lacunae within the scaffold (arrows) and collagen fibers are fully masked, closely resembling native cartilage matrix; *n* = 3. CO_2_‐lasered cartilage samples seeded with the same cell types are shown for comparison, indicating similar cell behavior in terms of differentiation. In addition, Collagen type II immunostaining of a commercially available Collagen I/III scaffold is presented, its loose meshwork deformed by the contractile forces of undifferentiated ASC. Native articular cartilage is included as a positive control, depicting the dense Collagen type II matrix that our approach aims to restore.

Histological examination after the cultivation period revealed the incisions to be densely filled with cells and newly deposited matrix that was well integrated with the scaffold (Figure 5). Moreover, hAC repopulated empty lacunae that were connected to the laser‐engraved surfaces. In the coculture group, as well as the ASC group, scaffold contraction occurred, suggesting the presence of cells containing a relevant amount of actin stress fibers, an indicator for an at least partly undifferentiated state of the cells. ASC generally remained undifferentiated despite the chondrogenic stimuli, producing neither GAG nor Collagen type II. In the coculture and chondrocyte groups, de novo tissue was rich in these characteristic cartilage components, indicating the presence of fully differentiated chondrogenic cells. While single collagen fibers were still distinguishable in the matrix produced by cells in coculture, hAC alone formed a homogenous tissue very similar to the fully masked collagen in native cartilage.

A comparison with CO_2_‐lasered scaffolds seeded with the same cell types highlights that cells on both decellularized cartilage scaffolds behave similarly in terms of differentiation as well as contraction in the ASC group, but the distribution of cells and de novo matrix is more homogenous on the femtosecond‐lasered scaffolds. A commercially available Collagen I/III scaffold, seeded with ASC, also allows for successful repopulation, but the loose matrix does not resemble native articular cartilage in collagen type or architecture, and is easily deformed by the contractile forces of undifferentiated ASC.

### 3.6. Osteochondral Plug Model In Vivo

To assess the behavior of cells on femtosecond laser–engraved scaffolds in a cartilage defect environment and get first impressions about the performance in vivo, reseeded scaffolds were implanted into artificial defects of osteochondral plugs and kept subcutaneously in an unloaded ectopic mouse model. ASC were precultivated outside of the defect for 3 weeks (2 weeks thereof under chondrogenic conditions), while hAC and cells in coculture were seeded in a one‐step procedure during implantation into the simulated defect.

After 6 weeks of subcutaneous cultivation, the scaffolds were well integrated into the defect and formed a continuous surface with the plug cartilage, complete with a superficial layer in the unlasered scaffold backbone. Scaffolds of all groups retained their shape during the cultivation period, without the contraction previously observed in the in vitro experiment, and cells had repopulated the laser‐engraved incisions. Due to cell proliferation during the precultivation period, ASC‐seeded scaffolds were wrapped in a thick cell layer, which nevertheless did not hamper implant integration to the adjacent cartilage. Scaffolds seeded with cells in coculture or hAC were covered by a thin cell layer on the cartilage surface. Spaces between the scaffold and the defect bottom were populated with cells (Figure 6).

Figure 6Scaffold integration and chondrogenic differentiation inside an artificial defect cultivated subcutaneously in a nude mouse model for 6 weeks. (a) Scaffolds of all groups were well integrated into the defects; due to the precultivation period before implantation, a dense cell layer surrounded ASC‐seeded scaffolds; a “tip effect” where Collagen type II expression is strongest deep inside the incisions was witnessed in the ASC and coculture group. (b) Cells not only filled the incisions but also had migrated into the matrix and repopulated empty lacunae. (c) Chondrocytes produced the most well‐differentiated matrix (asterisk), in some areas hardly distinguishable from the scaffold matrix (star); *n* = 6. For comparison, CO2‐lasered scaffolds seeded with the same cell types are included (AZAN staining), illustrating similar integration behavior within the defect.

**Figure:**
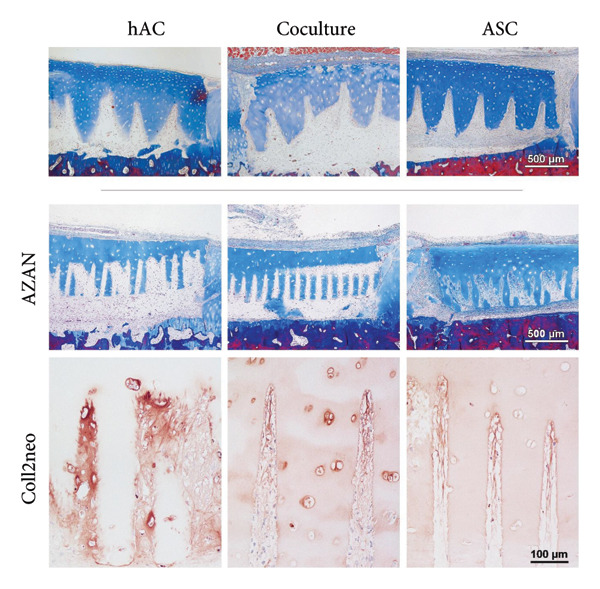
(a)

**Figure:**
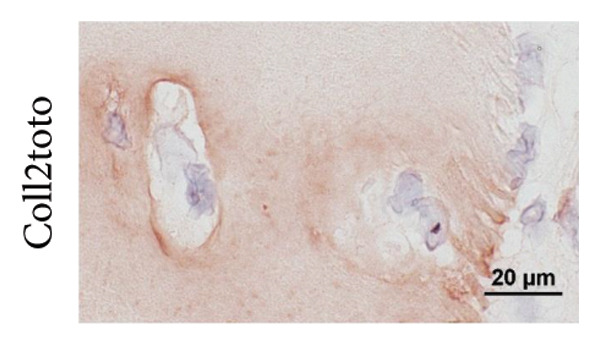
(b)

**Figure:**
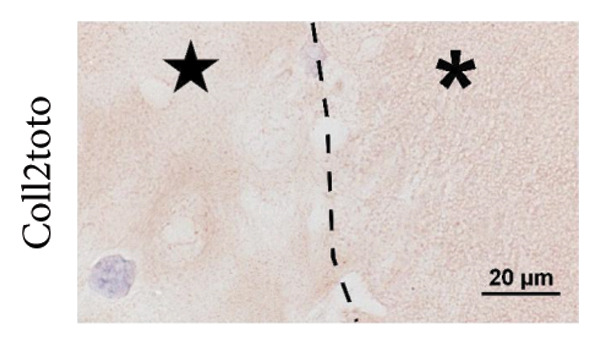
(c)

**Figure 7:**
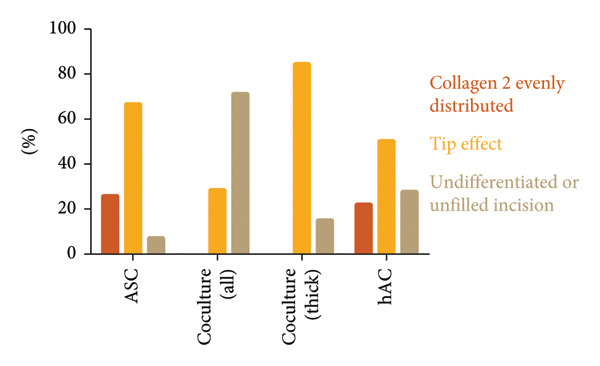
Patterns of Collagen type II distribution within femtosecond laser–engraved incisions. Scaffolds seeded with ASC showed Collagen type II deposition in nearly all incisions, with approximately two‐thirds displaying a characteristic “tip effect,” where differentiation was strongest at the incision tip. Coculture‐seeded scaffolds were highly heterogeneous: in thin samples, many incisions remained undifferentiated, whereas in thicker scaffolds, differentiation consistently presented with a tip effect. In hAC‐seeded scaffolds, Collagen type II was detected in three out of four incisions, most frequently with a tip effect pattern.

Immunohistochemistry revealed that the cells had started to deposit Collagen type II inside the incision. Immunohistochemistry revealed that the seeded cells had deposited Collagen type II inside the incisions, but with distinct distribution patterns depending on cell type and scaffold thickness (Figure 7). On ASC‐seeded scaffolds, Collagen type II was detected in almost all incisions, with two‐thirds (66%) showing a pronounced tip effect and 26% exhibiting even differentiation along the incision length. On scaffolds seeded with cells in coculture, differentiation was found to be highly dependent on scaffold thickness: in thin (600 μm) samples, the majority of incisions remained undifferentiated, while in thick (1 mm) scaffolds, Collagen type II was present in 85% of incisions, always with a tip effect. Chondrocyte‐seeded scaffolds displayed Collagen type II in 72% of incisions (22% were evenly differentiated and 50% showed a tip effect). In some places, the collagen fibers were already fully masked, as is the case in native cartilage. Cells and de novo matrix showed firm integration with the scaffold material, with collagen fibers stretching into the scaffold matrix via microfissures along the laser‐generated interface. Collagen type II–secreting cells were also found in empty lacunae at distances of up to 100 μm from the laser‐engraved edges even without any obvious connection to the incision space (Figure 6(b)).

Collagen fiber bundles produced by ASC and cells in coculture were aligned predominantly along the incisions, perpendicular to the surface, thus mimicking the orientation in the deep zone of native articular cartilage. The dense de novo matrix in chondrocyte‐seeded incisions closely resembled the scaffold matrix in its homogenous structure and Collagen type II staining (Figure 6(c)) and did not allow for a clear identification of a sharp border. In areas where collagen was not yet fully masked and single fibers were discernible, fibers appeared to have the same tendency for vertical alignment.

**Figure 9:**
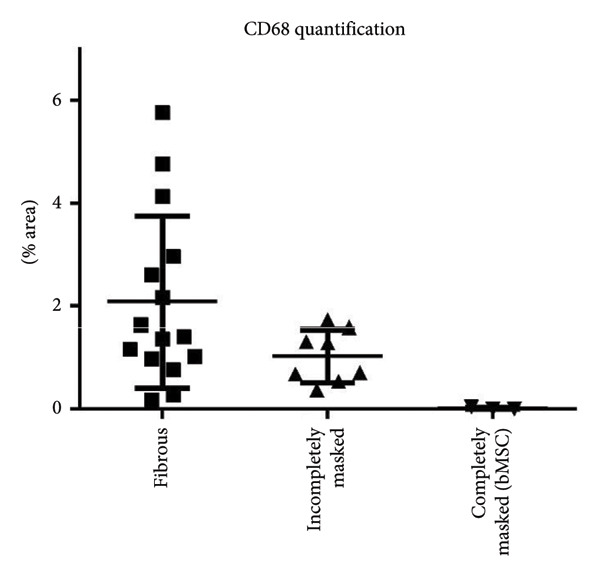
Quantification of macrophages within the laser‐engraved structures after cultivation within the artificial defect in a nude mouse model. A correlation became visible between the presence of macrophages and the stage of matrix formation within the incisions. Among incisions where single fibers were still clearly visible, many had a high number of macrophages (up to 5.7% of the area, as seen on a cross‐section in histology). As incisions get more densely packed, macrophages disappear. In the incisions that were filled with bone‐marrow–derived matrix, which might be assumed to be rich in macrophages, no CD68‐positive cells could be found.

Since several defects were open for cell invasion from the subchondral space, the presence of macrophages from the bone marrow was examined. Macrophages have been found in a majority of defects, identified via their immunoreactivity toward CD68. Their presence seemed to correlate inversely with the presence of a dense Collagen type II matrix (Figures 8 and 9). While there were plenty of macrophages in incisions where Collagen type II matrix was just being formed (2.1 ± 1.6% of the incision area positive for CD68), a lower number of macrophages (1 ± 0.5%) was found in incisions where Collagen type II expression was stronger and fibers were densely packed. In three cases (one from the ASC group and two from the coculture group), the subchondral bone was disrupted more severely, and bone marrow had invaded the defect. These incisions were densely filled with chondrogenic tissue, strongly stained for Collagen type II and devoid of CD68‐positive cells (only one incision tested positive, with 0.04% of the area). No significant differences could be demonstrated due to the low number of replicates (in the bone marrow–filled incisions group) or the large variations (within the other groups).

Figure 8Macrophages are present within the defect unless it is filled with Collagen type II matrix. (a) Bone‐marrow–derived cells entered the defect area from the subchondral bone via gaps (asterisk) created during defect preparation (seen here in a defect from the coculture group). (b) Macrophages, identified via their immunoreactivity toward CD68, were found in a majority of defects, especially those with incomplete differentiation of the new repair tissue. In scaffolds whose incisions were more densely filled with Collagen type II, the number of macrophages appeared lower. In two defects, a large amount of bone marrow invaded from the subchondral bone and the incisions as well as the surrounding spaces were densely packed with matrix positive for Collagen type II. No macrophages were observed in these defects.

**Figure:**
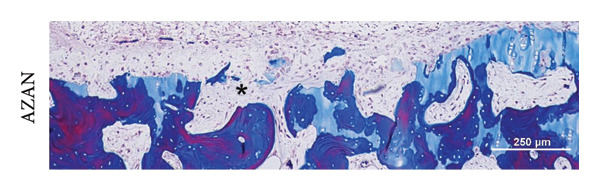
(a)

**Figure:**
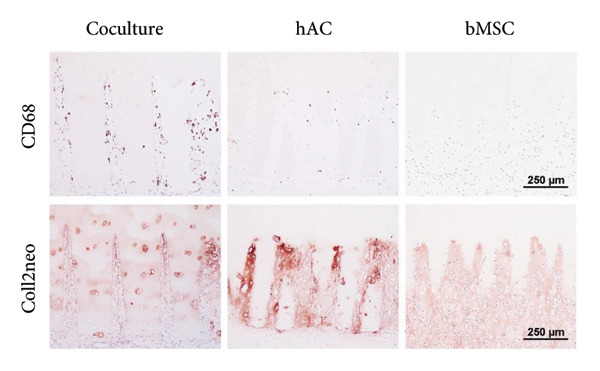
(b)

## 4. Discussion

This study aimed to evaluate whether femtosecond laser engraving can be used to precisely structure decellularized human articular cartilage in a way that promotes cellular repopulation and tissue regeneration, without compromising matrix integrity.

Decellularized articular cartilage of human origin represents the most homologous biomaterial for the treatment of articular cartilage defects, yet its full repopulation has never been achieved without providing the cells with entrance points and paths into the exceptionally dense matrix. In our previous study [29], we presented the concept of laser‐engraved linear incisions, promoting cellular repopulation of the cartilage matrix even deep within the scaffold, while leaving the bulk matrix and superficial zone intact. Within this study, using a more sophisticated laser technique, we were able to refine these scaffolds toward even finer structures.

Narrowly spaced incisions make for an optimal exchange of nutrients and stimulants between the cartilage environment, the scaffold, and the reseeded cells. The high precision of the femtosecond laser allows for narrow spacing between the engraved lines. Compared to CO_2_ laser engraving, the spacing could be reduced by half. The resulting thickness of the remaining structures (150–250 μm) resembles the chondron spacing naturally found in human articular cartilage. Moreover, after 6 weeks, cells have been found to not only fill the incisions but also repopulate empty lacunae about 50 μm deep into the scaffold. Notably, although both laser types allowed similar ingrowth depths, the greater density of incisions with femtosecond engraving results in a higher total scaffold volume being repopulated. When not laser engraved, the decell‐deGAG process, adapted for reduced treatment duration from our previously described protocol [16], left the scaffolds with a DNA content above the commonly accepted threshold for decellularized materials (50 ng/mg) [14]. Laser‐engraved cartilage, with its fine structures, was more easily decellularized, resulting in a DNA content below 30 ng/mg, lower than in CO_2_‐lasered scaffolds due to the thinner structures feasible with femtosecond laser engraving.

Another advantage of the femtosecond laser over the CO_2_ laser is the absence of thermal Collagen type II alterations that have been observed during our previous study [29]. In CO_2_‐engraved samples, Collagen type II immunoreactivity was diminished within ∼50 μm of the incision interface, indicating matrix denaturation due to thermal effects. All femtosecond laser generated surfaces feature Collagen type II with fully intact immunoreactivity. Superficial microfissures were observed on some samples, but have not demonstrated the negative effects of cell adhesion and may even be of advantage, as they provide a rough surface for the cellular attachment. Matric deposition within these fissures, as seen in histology, supports this suggestion and gives rise to the expectation of enhanced integration of scaffold and de novo matrix. Together with empty lacunae close to the surface, fissures further might aid in cellular infiltration. Matrix metalloproteinases (MMPs) might also play a role in this process, as they are, in small amounts, produced by all chondrocytes as part of natural cartilage turnover [51].

Different types of cells were included in this study to demonstrate the biocompatibility and chondrosupportive properties of the femtosecond laser incisions. While chondrocytes are the current clinical standard for matrix‐assisted repair of large cartilage defects, ASCs were added to the study as an alternative cell source to avoid drawbacks related to chondrocytes such as limited availability, donor site morbidity, and dedifferentiation during in vitro expansion. ASCs as therapeutically relevant mesenchymal stem cell types are known to be able to form functional cartilage in vitro and in vivo [52–55] and recently have achieved promising results in clinical studies regarding osteoarthritis [56–60] due to their anti‐inflammatory properties. hAC and ASC have been used as single cultures (to determine which effects are to be attributed to the respective cell type) as well as in coculture as the most promising future clinical application option. Replacing part of the hAC by ASC in a one‐step procedure with intraoperative cell harvest reduces adverse side effects associated with chondrocyte harvest and expansion and provides a faster treatment to the patient. Several studies have demonstrated that a majority of chondrocytes can be replaced by ASC with equal or even enhanced cartilage matrix production and stability [61–65]. There is an ongoing discussion on whether ASCs contribute to the coculture by differentiating into the chondrogenic lineage themselves or by enhancing the performance of chondrocytes via paracrine factors [66]. Pleumeekers et al. have observed that cartilage matrix production in a coculture model was performed entirely by chondrocytes [65], underlining the latter hypothesis. In our study, we were able to observe this beneficial effect only to a limited extent. During the in vitro reseeding tests, Collagen type II expression was absent in the ASC group, while cells were well differentiated in the coculture group, thus demonstrating some improvement. Both were, however, outperformed by the chondrocyte monoculture, leading to the assumption that de novo cartilage in the coculture is mainly produced by chondrocytes. This corresponds with the findings of Pleumeekers et al., who demonstrated that medium conditioned by MSC enhances the chondrogenic performance of chondrocytes, but chondrocyte conditioned medium does not have the same effect on MSC [65]. Further studies will have to be performed to determine whether ASC had any beneficial effect, whether they persist on the scaffold long term, and which part they played in matrix synthesis.

Chondrocytes, despite originating from donors of advanced age, gave the best results both in vitro as well as within the defect in vivo, demonstrating that the scaffold itself provides an excellent chondrogenic niche without the need for added growth factors.

ASC, which remained undifferentiated in the in vitro setting, performed better in the artificial defect model in vivo, despite the absence of added growth factors. This highlights the influence of a cartilage defect environment on the fate of implanted cells. Hypoxia in the subcutaneously implanted defect might have been of advantage, as a hypoxic environment is known to stimulate chondrogenic differentiation [67–69] and preserve the chondrogenic phenotype [70–72]. Still, the ectopic model does not yet fully mimic the environment or loading present in the joint. The presence of growth factors in the synovial fluid [73, 74] as well as the beneficial effect of loading on chondrogenic differentiation [75–78] might further enhance their performance in an orthotopic model.

While cells were generally best differentiated within the incisions, in groups with not yet fully completed chondrogenic differentiation, a “tip effect” was witnessed, corresponding to the observations made on CO_2_ laser–engraved scaffolds [29]. Chondrogenic differentiation appeared to start at the deepest point within the incision: the tip. In this area, cells are in closest contact with the scaffold matrix and therefore benefit strongest from the biochemical and spatial cues provided by the homologous material. Matrix components as well as articular cartilage matrix as a whole have been described to have chondroinductive effects [11, 12]. Moreover, cells and newly formed matrix quickly take up the space deep inside the incisions, potentially enhancing the chondrogenic stimulus by mimicking the confined environment present in hyaline cartilage. Hypoxia, again, might also play a role. Most interestingly, in the coculture group, the tip effect only existed in thick scaffolds (1 mm), which had deeper incisions. This might point out hypoxia as one of the key factors of a chondrogenic environment, yet it is unclear why the effect was limited to the co‐culture group.

Not only the amount but also the microarchitecture of newly formed Collagen type 2 stands in favor of the narrow incisions. In areas that were not yet fully filled with de novo cartilage matrix, the alignment of collagen fibers was discernible along the laser engraved edges, perpendicular to the cartilage superficial zone. This corresponds to the alignment naturally found in the deep zone of articular cartilage [79, 80], where the incisions are located. The collagen alignment is of particular importance for the long‐term functionality of regenerated articular cartilage. It determines microfluidics within the matrix and substantially contributes to the load‐bearing capacity of the tissue [81, 82].

While immunological responses are indeed important for scaffold integration, their relevance differs by anatomical context. In contrast to other types such as auricular or tracheal cartilage, articular cartilage lacks a perichondrium and is not in direct contact with epithelial tissues. Therefore, the type of scaffold–host interactions observed in airway models [83, 84] is not expected here. Instead, our study focused on macrophage presence within the defect area, which can reflect the local immune environment in articular cartilage repair. Immunostaining for CD68 revealed the presence of macrophages especially in areas where differentiation was yet incomplete. In fully differentiated sites, no CD68‐positive cells were observed. The exclusion of macrophages during the formation of de novo cartilage has already been observed before [85, 86] and might either indicate cartilage immunoprivilege [87] or simply be the consequence of an increasingly macrophage‐unfavorable environment in the dense repair tissue. Further studies in orthotopic models will be needed to evaluate long‐term immunological responses more comprehensively.

Our findings position femtosecond laser–structured cartilage as a promising off‐the‐shelf scaffold material that combines native‐like matrix composition with modern microstructuring techniques to support high‐quality cartilage regeneration. Yet, while femtosecond laser engraving offers distinct biological and structural advantages over CO_2_ laser engraving, it also comes with notable limitations. Femtosecond systems are considerably more costly, require specialized training for operation, and the engraving process itself is comparatively time‐consuming. In contrast, CO_2_ lasers are technically less demanding and allow for faster processing, which makes them more accessible for broader clinical translation. Thus, the choice of laser system will ultimately depend not only on biological outcomes but also on practical considerations of feasibility and scalability.

## 5. Conclusion

The hereby developed scaffold features, to our knowledge, the finest incisions in cartilage to have been achieved via laser engraving. It offers a defect filling that represents the native collagen architecture of articular cartilage, with the superficial layer remaining intact and undisturbed by laser engraving or matrix pretreatment [16]. Thus, the scaffold itself already serves as a high‐quality filling material for chondral defects, even before seeded cells have performed their work in scaffold remodeling and producing repair tissue. The engraved incisions enable the repopulation with therapeutically relevant cells and encourage chondrogenic differentiation as well as the orientation of newly produced collagen fibers in a way that mimics the architecture of native articular cartilage. In some areas, the de novo matrix was structurally indistinguishable from native hyaline cartilage already after 6 weeks. The short period of in vivo cultivation, together with the unloaded model, is the main limitation of this study, merely serving to show the potential of our approach. A long‐term orthotopic model is the next step in demonstrating the potential of laser‐engraved decellularized cartilage. The formation of high‐quality repair tissue by guided tissue formation and integration of a cartilage‐derived scaffold matrix with the neo‐cartilage could be the key to improving cartilage defect treatment, reducing long‐term graft failures, and providing great benefits for the patients as well as the healthcare system.

## Ethics Statement

The experimental protocols for the in vivo experiments were approved by the City Government of Vienna (Animal Use Permit No: MA58/982788/2015/13) prior to the study, in accordance with the Austrian law and the Guide for the Care and Use of Laboratory Animals as defined by the National Institute of Health (revised 2011).

## Consent

Human articular cartilage as well as articular chondrocytes (hAC) were gained from femoral heads of donors undergoing total hip arthroplasty or hemiarthroplasty, with written consent and the approval of the local ethical board.

## Conflicts of Interest

The authors declare no conflicts of interest.

## Funding

This work was funded by the Austrian Research Promotion Agency FFG (“CartiScaff” #842455), the Lorenz Böhler Fonds (16/13), and the City of Vienna project ImmunTissue (MA23 #30‐11).

## Supporting Information

Supporting Figure 1: 3D rendering of the μCT scan displaying the final scaffold. The dynamic 3D visualization of the scaffold’s structure emphasizes the fine incisions and pillar structure observed via μCT imaging. The video highlights the scaffold’s highly regular topography, created by orthogonally engraved lines that form regular pillars (provided as a separate file within the review system, labeled as “supporting_video_1”)

## Supporting information


**Supporting Information** Additional supporting information can be found online in the Supporting Information section.

## Data Availability

The data that support the findings of this study are available from the corresponding author upon reasonable request.
